# miR-6315 silencing protects against spinal cord injury through the Smo and anti-ferroptosis pathway

**DOI:** 10.1042/BSR20230030

**Published:** 2023-04-06

**Authors:** Zheng Ma, Yan Fan, Yufang Peng, Ligong Bian, Jianping Zhou, Lijuan Wang, Yan Xia, Sili Zheng, Yanlian Ji, Yanbing Han, Chengan Feng, Yingchun Ba

**Affiliations:** 1 Department of Anatomy and Histology and Embryology, Faculty of Basic Medical Science, Kunming Medical University, Kunming 650500, Yunnan, China; 2 Department of Orthopaedics, Chenggong branch of Kunming Yan’an hospital, Kunming 650500, Yunnan, China; 3 Department of Anatomy, Haiyuan College, Kunming Medical University, Kunming 650106, Yunnan, China; 4 Department of Neurology, the First Affiliated Hospital of Kunming Medical University, Kunming 650032, Yunnan, China

**Keywords:** miR-6315, neural repair, primary spinal neurons, Smo, spinal cord injury

## Abstract

Spinal cord injury (SCI) causes permanent damage and has a high disability rate. Currently, no efficient therapeutic strategy is available for SCI. The present study investigated the mechanisms of microRNAs (miRNAs) in rats with spinal cord injury. Whole transcriptome sequencing (WTS) was used for analyzing miRNA and messenger RNA (mRNA) expression patterns in rat spinal cord tissue at different time points after SCI. Gene Ontology (GO) and KEGG pathways were analyzed to obtain crucial functional pathways. miR-6315 was the most significantly up-regulated and differentially expressed miRNA after 24 h of SCI; the expression of miR-6315 gradually decreased after 3 and 7 days of SCI. Bioinformatics analysis was conducted to predict the targeting relation of miR-6315 with Smo, and qRT-PCR and dual-luciferase reporter assays were conducted for verification. The miR-6315 silencing (miR-6315-si) adenovirus was successfully constructed. miR-6315 knockdown treatment significantly promoted functional behavioral recovery in rats post-SCI through using Basso–Beattie–Bresnahan (BBB) locomotor rating scale and the inclined plane test. The neuronal axon regeneration and neuronal migration were promoted, and cell apoptosis was attenuated in treated SCI rats and Glu-treated neurons after miR-6315 knockdown using immunofluorescence and scratch assays. We discovered that Smo and anti-ferroptosis pathway factors, xCT, GSH, and GPX4, may be involved in miR-6315-regulated SCI repair. The expression of miR-6315 was negatively correlated with Smo, xCT, GSH, and GPX4. In conclusion, miR-6315 may be a potential target in the treatment of SCI.

## Introduction

Spinal cord injuries (SCIs) represent structural and functional injuries due to direct or indirect violence, which causes autonomic, sensory, and motor dysfunction underneath the injury level [1–3]. The scope and clinical presentations of SCI are determined by the primary nature and position of the injury. Complete SCI, one of the most severe SCIs, is the total loss of sensory and motor functions underneath the spinal cord level due to spinal cord transection (SCT), with poor regeneration ability. Once a complete injury occurs, there is no cure [4,5]. SCI treatment remains a challenge to the medical community [6–8] because of the extremely weak regeneration capacity of the spinal cord.

Studies have demonstrated that the mechanism leading to the termination of the initial axonal regeneration activity after SCI remains unclear [9,10]. Some scholars believe that the termination of regenerative activities may be related to a microenvironment that inhibits growth cones. The pathogenesis of SCI is complex and is classified as primary and secondary injuries [11,12]. The primary SCI occurs passively immediately following injury (usually within 4 h) and is irreversible. The secondary SCI is gradually formed within minutes to several days following the primary injury [7,13] and is accompanied by diverse intracellular metabolic and genetic alterations, including spinal cord tissue edema, oxygen free radical damage, inflammation, axonal demyelination, destruction of blood–spinal cord barrier, cell apoptosis, and necrosis [14,15]. Therefore, the strategy of enhancing the injured spinal cord microenvironment for minimizing secondary injury should be adopted for treating SCIs; this can inhibit further neuronal loss and stimulate axonal regeneration. However, the mechanisms that improve the SCI microenvironment remain unclear [3]. Further studies are required to elucidate the mechanism and identify effective neurotherapeutic targets for SCI.

In recent years, several findings have suggested that RNAs with diverse lengths interact with each other, such as the interactions between long noncoding RNA (lncRNA) and microRNA (miRNA), miRNA and mRNA, LncRNA and mRNA, forming a mutual regulatory network of lncRNA–miRNA–mRNA [16–18]. MiRNAs, the endogenous noncoding small RNAs, are approximately 20–23 nucleotides in length and are extensively distributed in various body fluid and tissue types [17,19], such as the brain, spinal cord, serum, urine, and cerebrospinal fluid. They exert critical effects on neurodevelopment, neurological diseases, as well as neurodegenerative diseases [20–22]. Mature miRNAs negatively affect the post-transcriptional regulation of genes by forming complementary pairs with target mRNAs, resulting in mRNA translational repression or degradation [23]. Although the relationship between the miRNAs and mRNAs is well-investigated, the regulatory relationship between miRNAs and target mRNAs in the spinal cord post-SCI remains unclear [24]. Therefore, a comprehensive analysis of miRNAs and mRNAs to analyze the miRNA–mRNA axis within SCI must be performed.

This study conducted whole transcriptome sequencing (WTS) to reveal a significant up-regulation in the miR-6315 level; moreover, its expression reached the highest level after 24 h of SCI and gradually decreased after 3 and 7 days of SCI. Bioinformatics prediction and dual-luciferase reporter gene detection revealed that miR-6315 has a site regulatory relationship with Smoothened (Smo). The GeneMANIA database was used to identify Smo targets based on co-expression profiles; the results revealed that the downstream signaling pathway factor Smo might regulate the anti-ferroptosis pathway factor xCT. To better understand the potential role of the miR-6315/Smo axis in subacute neurological injury after SCI, we further demonstrated their functions and mechanisms by using adenovirus techniques. We demonstrated that miR-6315 affects motor function recovery, neuron survival, apoptosis, and ferroptosis post-SCI, and its mechanism was related to the regulation of Smo and anti-ferroptosis pathways. In summary, this work illustrated the novel pluripotent regulation axis through miR-6315-mediated Smo.

## Materials and methods

### Animals and experimental design

Normal female specific pathogen-free (SPF) Sprague-Dawley (SD) rats, each weighing 180–220 g, were provided by the Experimental Animal Center of Kunming Medical University. All of the assays were conducted following Guide for the Care and Use of Laboratory Animals from US National Institutes of Health. The animal study protocols were reviewed and gained approval from Animal Care and Welfare Committee of Kunming Medical University (approval No.: kmmu 20211409). All animal experiments took place in the Basic Medicine Building of Kunming Medical University. Each rat was housed in a single cage in a clean-grade animal room with a 12-h light/dark cycle, room temperature of 25°C, and free access to adequate food and water.

To identify the SCI rat model, Nissl staining and behavioral experiments were performed to evaluate neuronal morphology and motor recovery in rats. Every injury type contained 2 groups (*n*=6), namely, Sham and 1 d survival. To determine the effect of miR-6315 on the subacute phase repair of rat SCI and the underlying mechanism, 4 groups were used on day 7 post-SCI, namely sham, SCI, SCI + NC, and SCI + miR-6315-si. Neuronal morphology was examined using immunohistochemistry (IHC) and immunofluorescence (IF) assays (*n*=6). Ferroptosis markers and axonal growth cone pathway factor Smo were measured using real-time PCR (RT-PCR) and Western blot (WB) assays (*n* = 3/group). Three rats were randomly selected for the behavioral test to evaluate their motor recovery. At the end of the experimental period, for IHC and Nissl staining, rats put into deep anesthesia by tail-vein injection of high dose 2% (w/v) pentobarbital (120 mg/kg), 0.9% normal saline and 4% paraformaldehyde (PFA) were added to perfuse the hearts in succession. Spinal cord tissues were subjected to overnight fixation with 4% PFA. For RT-PCR and WB experiments, rats put into deep anesthesia by tail-vein injection of high dose 2% (w/v) pentobarbital (120 mg/kg), 0.9% saline was added to perfuse the hearts, and the spinal cord tissues were removed and stored in liquid nitrogen quickly.

### Bioinformatics analysis

To extract total RNA, the spinal cord was dissected at the center of the lesion at 1, 3, or 7 days post-SCI. Differentially expressed mRNAs (DE mRNAs) and miRNAs (DE miRNAs) in the spinal cord tissue were screened using whole transcriptome sequencing. In addition, the Gene Ontology (GO) and Kyoto Encyclopedia of Genes and Genomes (KEGG) analyses were conducted using the enrichment analysis tool DAVID. The Miranda software and the Ensembl database were used to predict 3′-untranslated regions (UTRs) of mRNA and miRNA targets.

### Recombinant adenoviruses construction, transfection, and implantation

Recombinant adenoviruses expressing miR-6315 siRNA or scramble RNA (NC) were constructed by GeneChem (Shanghai, China). In cell experiments, adv-si-NC and adv-si-miR-6315 were diluted in a neuron-specific or basal medium and administered at a concentration of 100/well (multiplicity of infection [MOI]). To construct the *in vitro* SCI model, the spinal cord neurons and PC12 cells were stimulated to induce an injury 24 h after the adenovirus infection in accordance with the aforementioned method. In animal experiments, adv-si-NC and adv-si-miR-6315 (1 µl, 1 × 10^10^ pfu/ml, *n*=6) were added to the injured spinal cord at a depth of 4 mm by using a microinjector (5–5 µl) in 10 min. The injections were administered at three injection sites at both upper and lower ends of spinal cord transection. The fascia, muscle, and skin were then sutured in sequence.

### Luciferase reporter assays

To verify the binding of miR-6315 to Smo 3′-UTR, a wild-type (WT) Smo 3′-UTR luciferase reporter vector, named wt-Smo, was constructed through the insertion of a Smo 3′-UTR fragment into a firefly luciferase vector (Genechem, China). The mutant Smo 3′-UTR vector was constructed through the mutation of the miR-6315 binding site at Smo 3′-UTR, and the vector was named mut-Smo. In addition, the Renilla luciferase vector was used as an internal control. miR-6315 mimics/inhibitor was cotransfected with the aforementioned vectors into PC12 cells. The Dual-Luciferase Reporter Assay System (Promega, U.S.A.) was employed to detect luciferase activities. Firefly fluorescence / renilla fluorescence values were considered the final relative luciferase activity.

### Spinal cord transection (SCT)

Rats were anesthetized via intraperitoneal administration of 2% (w/v) pentobarbital (40 mg/kg). Subsequently, a T8-T10 vertebral laminectomy was performed to expose the T10 spinal segment. A hook was passed from the spinal cord ventral side and was carefully lifted by 1 mm. Surgical scissors were used to sever the whole spinal cord, followed by hook removal. Adenovirus was injected at both upper and lower SCT ends, as previously described. Finally, the muscle, fascia, and skin were sutured in sequence. After surgery, the rats’ bladders were massaged, and they were manually urinated three times daily. The rats were injected intramuscularly with benzylpenicillin (dose 40 IU) daily for 5 days. The sham animals underwent identical laminectomy without spinal cord damage. Rats were euthanized if they exhibited very abnormal behavior after spinal cord injury, such as 40% weight loss, self-mutilation of lower extremities or abdomen resulting in inability to urinate, difficulty in breathing or very painful phenomena. Euthanasia was accomplished by over-anesthetizing the rat to death with a large dose of 2% (w/v) pentobarbital (200 mg/kg) administered through the tail vein.

### Basso–Beattie–Bresnahan (BBB) score

The 21-point Basso–Beattie–Bresnahan (BBB) score scale was used to evaluate the behavioral recovery of SCI rats, with a score of 0 indicating the absence of hindlimb movement and a score of 21 indicating normal movement. The rats were subjected to behavioral testing on 1, 3, and 7 days after surgery. The sensory and motor function of the rats were assessed in an open field by three researchers blinded to the experimental design. The final scores were the average of the scores obtained by three investigators.

### Inclined plate grasp tests

The weight-bearing ability of rat hindlimbs was determined using the inclined plate grasp test [25]. In brief, each rat was placed on a rubber pad-covered wooden board, with its body facing forward and at a perpendicular angle to the ground. The initial plate angle was 0° and was increased by 5° in each trial. The maximal angle at which a rat was able to stay for a 5-s period on the inclined plate was considered its functional score. The measurement time interval for each animal was 5 min, and each rat’s weight-bearing ability was measured five times.

### Footprint experiments

The rat’s gait behavior and motor coordination were evaluated using the footprint analysis. Rat forelimbs and hindlimbs were immersed in a black and red dye, respectively, and passed through a narrow point horizontal channel (1 m long, 7.5 cm wide) in a white paper. The rats were encouraged to walk directly to the finish line to obtain representative gait images and assess coordination. Evaluations were performed by two researchers without knowledge of the experimental design.

### Nissl staining

Nissl bodies were stained with Nissl stain to observe neuronal cell structure in spinal cord slices. In brief, this work processed spinal cord tissue through fixation, paraffin embedding, and slicing to 7 µm sagittal sections. The spinal cord slides were later subjected to 40 min staining in a 60°C incubator by using 1% toluidine blue (Beyotime, China). Five slices of spinal cord tissues were randomly selected for each animal, and the neuron number within spinal cord slices was determined using a light microscope (magnification: 200×, China). The ImageJ software (U.S.A.) was used for determining the neuron number within spinal cord slices.

### Cultures of primary spinal cord neuronal cells and PC12 cells

This work cultured primary spinal cord neuronal cells as per the protocol described in a study [26]. In brief, we used euthanasia by inhalation anesthetizing 1-day-old pups in a wide-mouthed bottle containing isoflurane (R510-22-10, RWD) and removing them for decapitation when they entered deep anesthesia. The dura and pia mater were gently stripped, and the spinal cord tissues were cut to 1 mm^3^ size. Subsequently, the tissue was digested with papain (Sigma, U.S.A.) to form a cell suspension. The cells were counted under a light microscope and seeded into a 6-well plate at 2 × 10^6^ cells/well, followed by the culture at 37°C, 95% saturated humidity, and 5% CO_2_ conditions. On day 2 of the culture, neural basal medium (Gibco, U.S.A.) containing 0.5 mM glutamine, 2% B27 (Gibco, U.S.A.), and 50 U/mL penicillin/streptomycin (PS, Hyclone, U.S.A.) was added to replace the original medium. On day 3 of the culture, neurons were infected with adenovirus as described in the aforementioned method. Transfection of the spinal cord neurons was determined by the presence of red fluorescence in inverted fluorescence microscopy. Five visual fields were randomly obtained for each sample, and the Image-Pro Plus 6.0 software was used to determine spinal cord neuronal cell number, neuron soma area, and the length of neuron axons.

PC12 cells (highly differentiated) were obtained from the American Type Culture Collection (ATCC, CRL-1721, U.S.A.) and were cultured in DMEM-F12 (Hyclone, U.S.A.) supplemented with 1% PS (Hyclone, U.S.A.) and 10% fetal bovine serum (FBS, Gibco, U.S.A.) at 5% CO_2_ and 37°C conditions. Cell passaging was performed when PC12 cells grew to approximately 80% of the bottom of the T25 cell culture flask. The medium of PC12 cells was poured off and 2 ml of pre-warmed PBS was added to wash the cells. Then, 2 ml of 0.25% trypsin (Thermo Fisher Scientific, U.S.A.) was added and digested at 37°C for 5 min. The cells were observed to be suspended under the light microscope (Olympus, Japan) and then the digestion was immediately discontinued with DMEM-F12 medium containing 10% FBS. The collected cell suspension was transferred to a 15 ml centrifuge tube and centrifuged at 111.8 × ***g***/min for 4 min. The supernatant was discarded and new medium was added to the cell sediment, and the cell suspension was transferred to a new culture flask or plate for culturing. PC12 cells were used for the identification of miR-6315-interfering adenovirus when they were passaged to the third generation.

### Scratch test

The migration ability of spinal cord neurons was determined using scratch experiments. Spinal cord neuronal cells (5 × 10^5^/well) were inoculated into 12-well plates with adenovirus, and a cross-scratch was performed using a 100-µl pipette tip. Subsequently, floating cells were completely removed by washing with DMEM-F12 medium three times, and the cells were cultured at 37°C. Images were collected after 0, 12, 24, 36, 48, 72, and 96 h by using an optical microscope. The Image-Pro Plus 6.0 software was used for the detection of scratch width. The scratch healing rate was determined as follows: [(scratch width at 0 h − scratch width at 24 h)/scratch width at 0 h] × 100%.

### Immunofluorescence staining

After rats were put into deep anesthesia by tail-vein injection of high dose pentobarbital, 0.9% normal saline and 4% paraformaldehyde (PFA) were added to perfuse the hearts in succession. Spinal cord tissues were subjected to overnight fixation with 4% PFA. Subsequently, the tissues were processed routinely, washed, dehydrated, cleared, dipped in wax, and embedded. The tissue immunofluorescence staining was performed by subjecting paraffin-embedded spinal cord tissues to deparaffinization and rehydration with graded ethanol. The tissues were washed three times with phosphate-buffered saline (PBS), and the antigen was retrieved from the sections with 0.01 mol/l citrate buffer, followed by 30-min blocking by using 5% goat serum at 37°C, and overnight primary antibody incubation at 4°C. Anti-NeuN (Abcam, ab104224, mouse, 1:200), anti-GAP43 (Abcam, ab75810, rabbit, 1:500), and anti-SYP (Proteintech, 17785-1-AP, rabbit, 1:400) were used as antibodies. Subsequently, appropriate fluorescence-conjugated secondary antibodies, including Alexa Fluor® 488 (Bioss, bs-0296G-AF488/ bs-0295G-AF488, Goat anti-rabbit/goat anti-mouse, 1: 200), were added to the sections, followed by further incubation in the dark for another 1 h at 37°C. 4′,6-diamidino-2-phenylindole (DAPI, Solarbio) containing anti-fluorescence quencher was added to stain the nuclei, and the sections were directly sealed with coverslips. An inverted fluorescence microscope (Zeiss, Primovert, Germany) was used for immunofluorescence (IF) analysis. The number of positive neurons of NeuN was counted at three randomly selected fields within each section by using the ImageJ software [26].

The IF staining was performed as per the protocol described in a study [26]. In brief, a solution containing 5% goat serum and 0.3% Triton X-100 was added to block spinal cord neurons, followed by overnight incubation with primary antibodies (anti-NeuN, anti-GAP43, and anti-SYP) at 4°C. The subsequent steps were the same as those used for the tissue samples. Finally, an inverted fluorescence microscope (Leica, Wetzlar, Germany) was used to obtain images from at least five pictures of the same area per group. The percentage of NeuN^+^/DAPI, GAP43^+^/DAPI, SYP^+^/DAPI was counted using the Image J software. Three neurons were randomly selected from at least 10 cells, and the average length and area of the neuronal axons and surfaces (cell bodies) were measured using the Image-Pro Plus 6.0 software.

### TUNEL assay

TUNEL assay was performed to evaluate the spinal cord tissue for neuronal apoptosis. In brief, primary spinal cord neuronal cells were inoculated into 6-well plates with three coverslips. Subsequently, TUNEL staining was performed using a One-step TUNEL Apoptosis In Situ Assay Kit, as per the manufacturer’s protocol (KeyGEN BioTECH, China). The cell samples were immersed in 1% Triton X-100 permeabilization solution at an ambient temperature for a 5-min period. Proteinase K working solution was added dropwise to the paraffin sections of the spinal cord tissues for 30 min at 37°C. The subsequent procedure was the same for cells and tissue samples. The TdT enzyme reaction solution and streptavidin-fluorescein reagent were used for staining and prepared according to the manufacturer’s instructions. Finally, the cells and tissue samples were stained with DAPI containing an anti-fluorescence quencher for 5 min at room temperature. Images from five different fields of each sample were captured using an inverted fluorescence microscope. The number of cells was measured using the ImageJ software, and the rate of apoptosis-TUNEL-positive neurons/total number of DAPI-labeled cells was calculated.

### Quantitative RT-PCR (qRT-PCR)

Total RNA was extracted from the spinal cord neurons (1 × 10^6^ cells/1 ml) and tissues (100 mg/1 ml) using RNAiso™ Plus (Takara, Japan) and 1 µg of total RNA was used to synthesize cDNA. The reverse transcription kit (Vazyme, China) was utilized for synthesizing cDNA under the following conditions: 15 min at 50°C, followed by 85°C for 5 s. The expression of total cDNA was measured using qRT-PCR with Taq Pro Universal SYBR qPCR Master Mix (Vazyme, China) and a qRT-PCR system (Thermo Fisher Scientific, DE); the total volume of q-PCR was 20 µl, including 10 µl of 2 × Taq Pro Universal SYBR qPCR Master Mix (Vazyme, China), 0.4 µl of each primer and 2 µl of cDNA diluted 5-fold; q-PCR was performed in a QuantStudio™ Real-Time PCR system with the following thermal cycling conditions: 30 s at 95°C followed by 40 cycles of 1 s at 95°C and 30 s at 60°C, and 95°C for 15 s, 60°C for 60 s, and 95°C for 15 s. The β-actin gene was used as the endogenous reference. For miRNA analysis, cDNA was prepared using a Bulge-Loop miRNA qRT-PCR Starter Kit (RiboBio, China), with U6 snRNA (RiboBio, China) being the endogenous reference. The 2-ΔΔCT approach was used to assess relative gene expression. The primer sets for Smo, xCT, GPX4, ACSL4, miR-6315, and β-actin genes are listed in Table 1.

**Table 1:** Primer sequences

Genes	Forward (5′-3′)	Reverse (5′-3′)
Smo	TCCCATGATGGACCTGTTGC	CAGGGGTGACAGACACATCC
xCT	CCATCATCATCGGCACCGTCATC	TACTCCACAGGCAGACCAGAACAC
GPX4	GCCGGCTACAATGTCAGGT	ACCACGCAGCCGTTCTTATC
ACSL4	TCGAAGCCGCACTGAAGAAT	CTACCCCCTTCTGTTGTGCC
miR-6315	CGTCTGGACAGGACAGGCC	AGTGCAGGGTCCGAGGTATT
β-Actin	GAAGATCAAGATCATTGCTCCT	TACTCCTGCTTGCTGATCCA

Abbreviations: ACSL4, acyl-CoA synthetase long-chain family member 4; GPX4, glutathione peroxidase 4; Smo, smoothened; xCT, xc- system.

### Glutathione (GSH) assay

The glutathione (GSH) contents in the spinal cord tissues and neuron lysates were assessed using a GSH (Solarbio, #BC1175) assay kit, according to the manufacturer’s instructions. GSH was precipitated from the incubated samples by using a lysate buffer. The extracted samples were measured using the Thermo Fisher Multiskan FC spectrophotometer (Thermo Fisher Scientific, DE, U.S.A.).

### WB assay

WB assay was performed as described in a study [26]. Proteins from spinal cord neurons were extracted, and 10% SDS-PAGE (Beyotime, China) was used to separate proteins from the supernatant containing 6 µg protein/10 µl. The proteins were then transferred to a nitrocellulose membrane (Millipore, U.S.A.) by using the semi-dry approach. Membranes were blocked using 5% defatted milk, followed by incubation with anti-ACSL4 antibody (Abcam, ab155282, rabbit, 1:10,000; U.S.A.), anti-xCT antibody (Novus Biologicals, NB300-318, rabbit, 1:1000; U.S.A.), anti-GPX4 antibody (Novus Biologicals, NBP2-75511, rabbit, 1:1000; U.S.A.), and anti-β-actin antibody (Abbkine, cat# A01010, mouse, 1:10,000) overnight at 4°C. Membranes were rinsed and incubated using suitable HRP-labeled goat anti-mouse (cat# A21020) and anti-rabbit (cat# A21010) IgG secondary antibodies (1:5000) at room temperature for 1 h and visualized using the chemiluminescent imaging system (Bio-Rad).

### Statistical analyses

SPSS 21.0 and GraphPad software 6.0 were employed for data analyses; data are presented as mean ± SD. In two-group comparisons, data were subjected to S-W (Shapiro–Wilk) and K-S (Kolmogorov–Smirnov) normality tests. Data with normal distribution were analyzed by a Student's *t*-test, and those with abnormal distribution were analyzed by a Mann–Whitney test. In multiple group comparisons, data were subjected to S-W and K-S normality tests. Data with normal distribution were analyzed by a one-way analysis of variance (ANOVA) plus multiple comparison test (Fisher’s protected least significant difference test, PLSD), and data with abnormal distribution were analyzed via a Kruskal–Wallis test. Statistical significance was marked with *, # *P*<0.05; **, ## *P*<0.01; ***, ### *P*<0.001.

## Results

### SCI rat model construction

**Figure 1:**
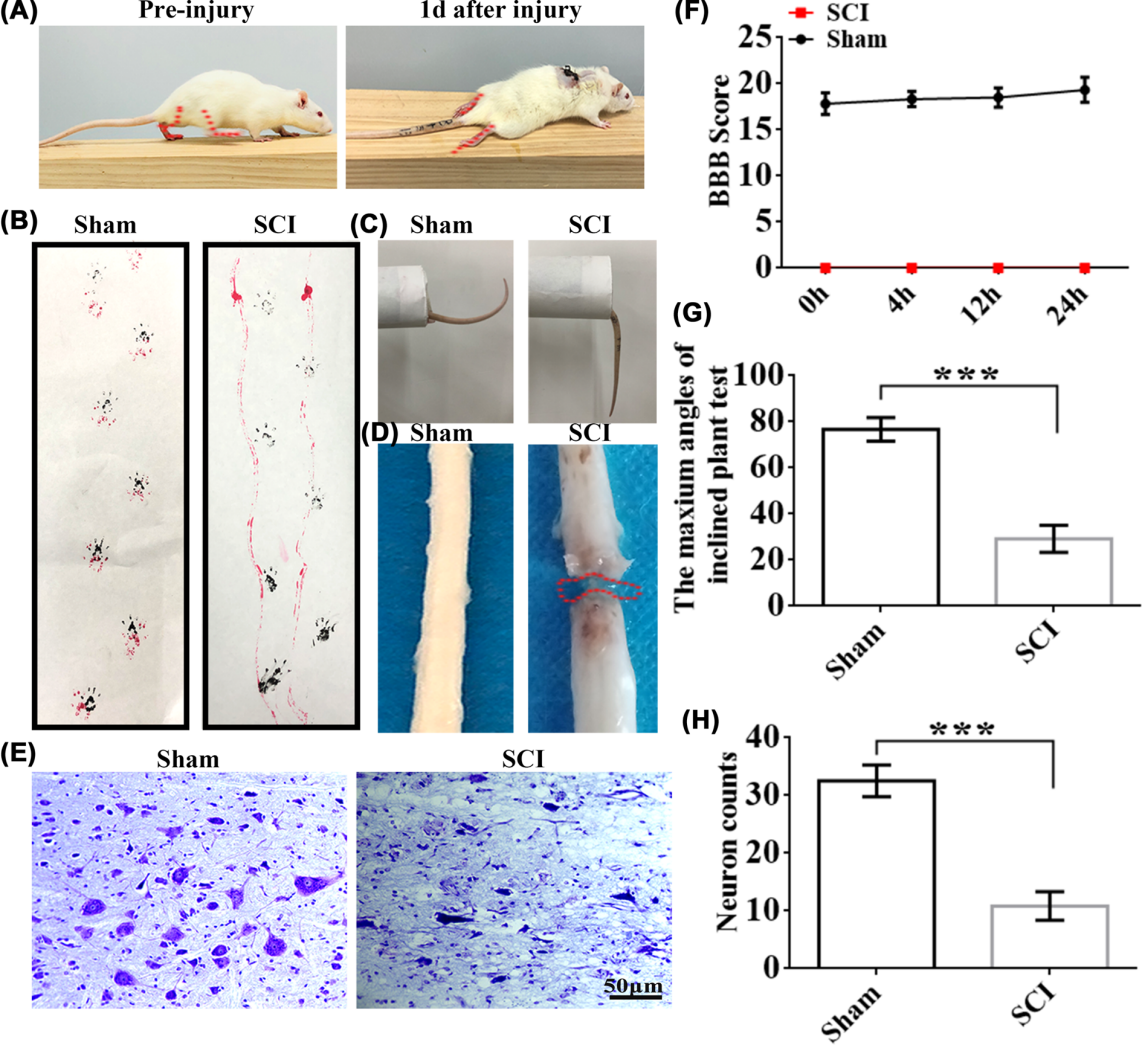
Assessment of locomotor recovery after SCI (**A**) Typical images showing rats walking during the SCI process. Red dotted lines represent the limb movement regarding the hip, knee, and ankle articulation. (**B**) Typical footprints for rat walking 1-day post-SCI. Black and red indicate front paw and hind paw prints, respectively. (**C**) Typical images highlighting the difference between Sham-treated rats and SCI-treated rats regarding the tail position. (**D**) Gross morphology of the spinal cord. The red dotted line represents the spinal cord injury center. (**E**) The spinal cord morphologic alterations in the Sham and SCI groups through Nissl staining. (**F**) BBB score quantification corresponding to rats treated with SCI and Sham (*n* = 6 each). (**G**) The quantitative analysis of the maximum angle of the inclined plate in the SCI and Sham groups (*n* = 6 each). (**H**) Quantitative analysis of total neuron counts in the spinal cord between the Sham and SCI groups (*n* = 6/group). Values represent mean ±S.D. ***P<0.001 vs. the Sham group.

To test new targets for promoting functional recovery following SCI, we used the complete SCT rat model. The rat hindlimbs were paralyzed immediately following complete SCT at the thoracic 10 levels. One day later, the lower body paralysis was confirmed by observing the posture of the rats, dragging the abdomen and tail during the movement, and confirming that the rats could not lift their tails (Figure 1A,C). To assess the hindlimb motor function, we performed a BBB test (BBB locomotor rating scale) at 0, 4, 12, and 24 h post-SCI, which presented a BBB score of 0 (Figure 1F). The observation of fore paw-hind paw movements by using footprint experiments also validated the BBB results (Figure 1B). The transection of the injured spinal cord was clearly visible in the gross morphology (Figure 1D). Thereafter, the hindlimb weight-bearing capacity of the rats was assessed using the inclined plate grasp assay. The results revealed that the maximum angle of the SCI group remarkably decreased in comparison with the Sham group (Figure 1G). The Nissl staining assay revealed that the Sham group exhibited normal spinal cord neuron morphology, and most cells in the SCI group had irregular morphology (Figure 1E). The quantitative analysis of the neurons indicated a decrease in total neuron number following SCI compared with the Sham group (Figure 1H).

### High-throughput sequencing (HTS) of genes, primary GO, KEGG pathways, and DE mRNAs and prediction of miRNA-mRNA binding sites

**Figure 2:**
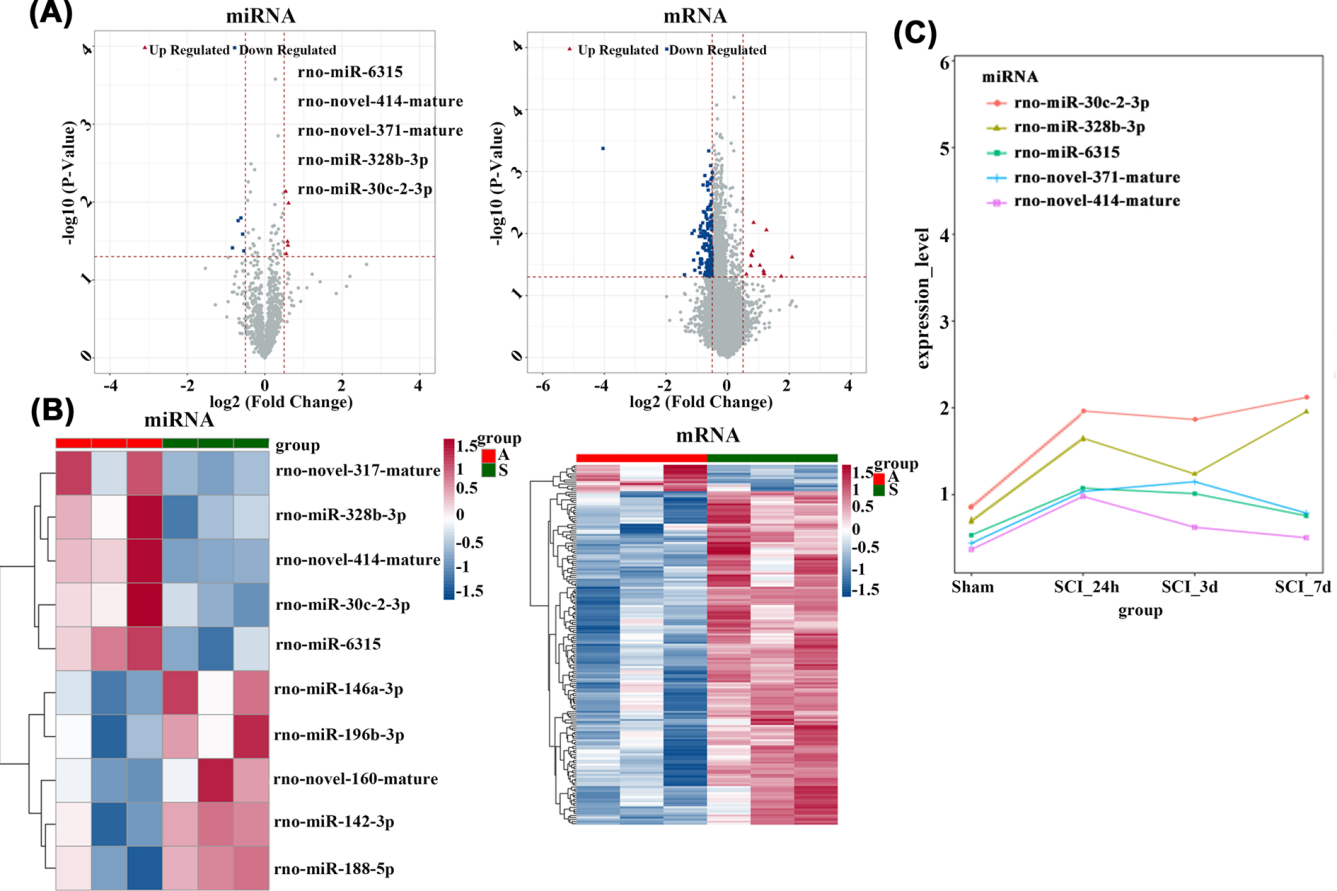
Gene HTS and DEmiRNAs (**A**) Volcano plots elucidate variance in DE miRNA (left) and DE mRNA (right) according to FC and *P* values. *X* and *Y* axes represent FC (log 2) and *P* values (−log 10). Red (FC > 2) and blue (FC < −2) points represent miRNAs or mRNAs with upregulation and downregulation, respectively, in the volcano plots. (**B**) The heatmaps revealing miRNA (left) and mRNA (right) determined through gene HTS at 24 h post-SCI. Blue pixels represent down-regulation, while red pixels represent up-regulation. A and S represent spinal cord tissue samples collected in SCI and Sham rats, respectively. (**C**) The expression curve of significantly up-regulated DE miRNAs over time. The *X*-axis represents 24 h, 3, and 7 days post-SCI, and *Y*-axis represents the expression level.

DE miRNAs and the coding genes within the spinal cord in SCI rats were examined at 24 h, 3 days, and 7 days through miRNA and mRNA sequencing. Compared with the Sham group, the expression of miRNAs and mRNAs in SCI rat spinal cord changed significantly (Figure 2A). The five miRNAs that were significantly upregulated were rno-miR-6315, rno-novel-414-mature, rno-novel-371-mature, rno-miR-328b-3p, and rno-miR-30c-2-3p. A volcano plot (Figure 2A) and heatmap (Figure 2B) were constructed to display DE mRNAs and DE miRNAs selected on the basis of fold changes (FC) and *P* values. To detect the expression changes of the significantly up-regulated miRNAs at 24 h, 3 d, and 7 d after SCI, we analyzed the expression levels of the significantly up-regulated DE miRNAs. The results revealed that the expression of miR-6315, with the most significant fold difference, reached the highest at 24 h after SCI and gradually decreased over time. Therefore, miR-6315 may be involved in the development of SCI (Figure 2C).

**Figure 3:**
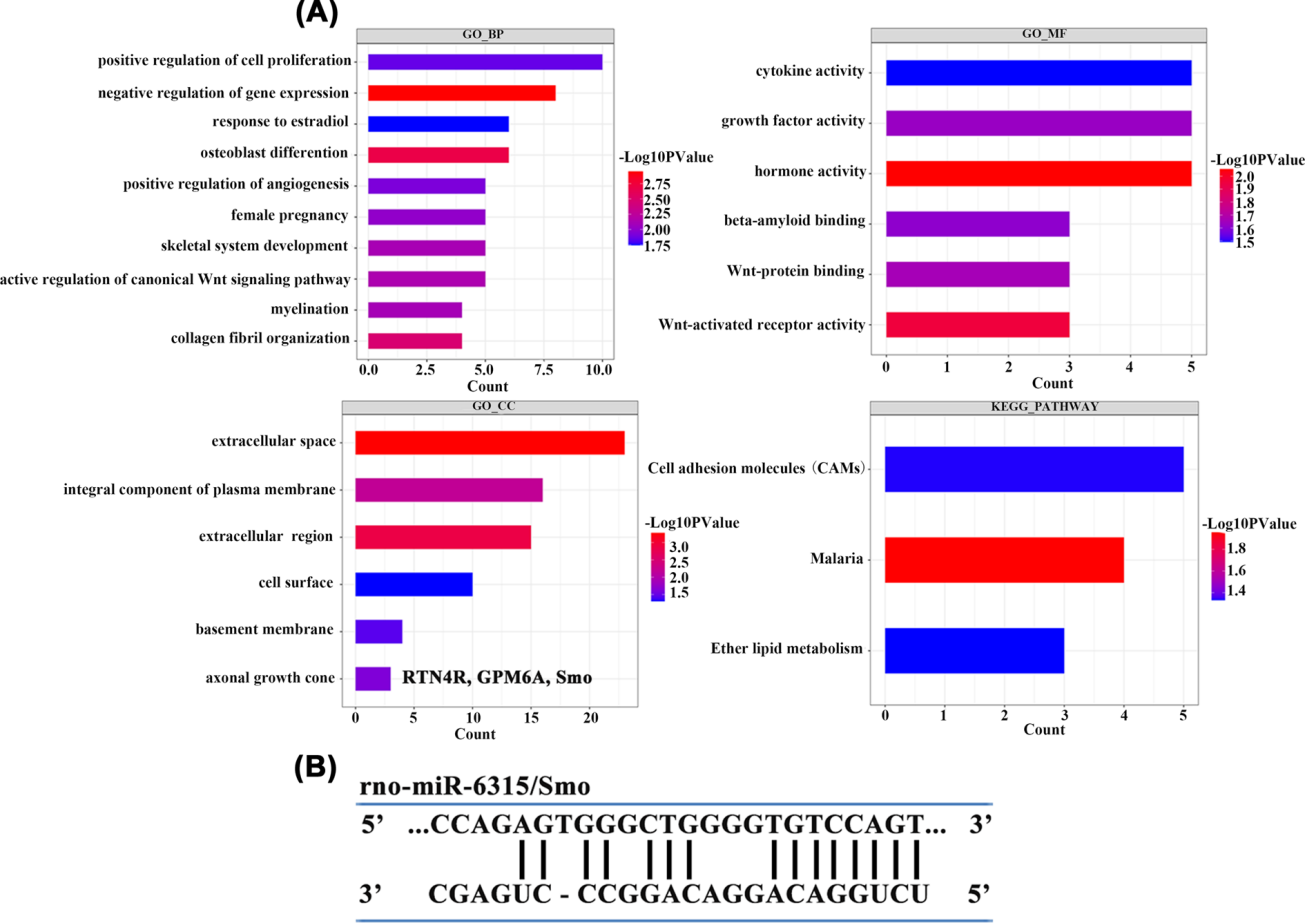
GO and KEGG analysis of DEmRNAs and miRNA-mRNA sequence binding (**A**) The *P* values of selected GO terms and their critical effects on SCI, with the lower p-value indicating a higher significance. Significance threshold *P*-value < 0.05 and the enrichment count (*Y*-axis) of at least 2 were regarded as significant enrichment results. The enrichment results in 32 GO BPs, 6 GO CCs, 6 GO MFs, and 3 KEGG pathways. (**B**) A predicted miR-6315/Smo target interaction axis after SCI.

On the basis of the DE mRNAs obtained by sequencing, we used the enrichment analysis tool DAVID to screen mRNAs with important functions in SCI. The significance threshold *P*-value of <0.05 and the enrichment count of at least two were regarded as significant enrichment results (Figure 3A). We analyzed the GO system involved in DE mRNAs and the KEGG pathway with a critical effect on SCI to further enrich the axonal growth cone pathway, including the gene sets RTN4R, GPM6A, and Smo (Figure 3A). We used the Ensembl database to screen three target gene sequences in the axonal growth cone pathway and the Miranda software to predict the binding sites between the three mRNAs in the pathway and significantly upregulated DE miRNAs. The results indicated that Smo has binding sites for miR-6315, and the sequence binding diagram is provided in Figure 3B.

### Verification of DEmiRNA and their interactions

**Figure 4:**
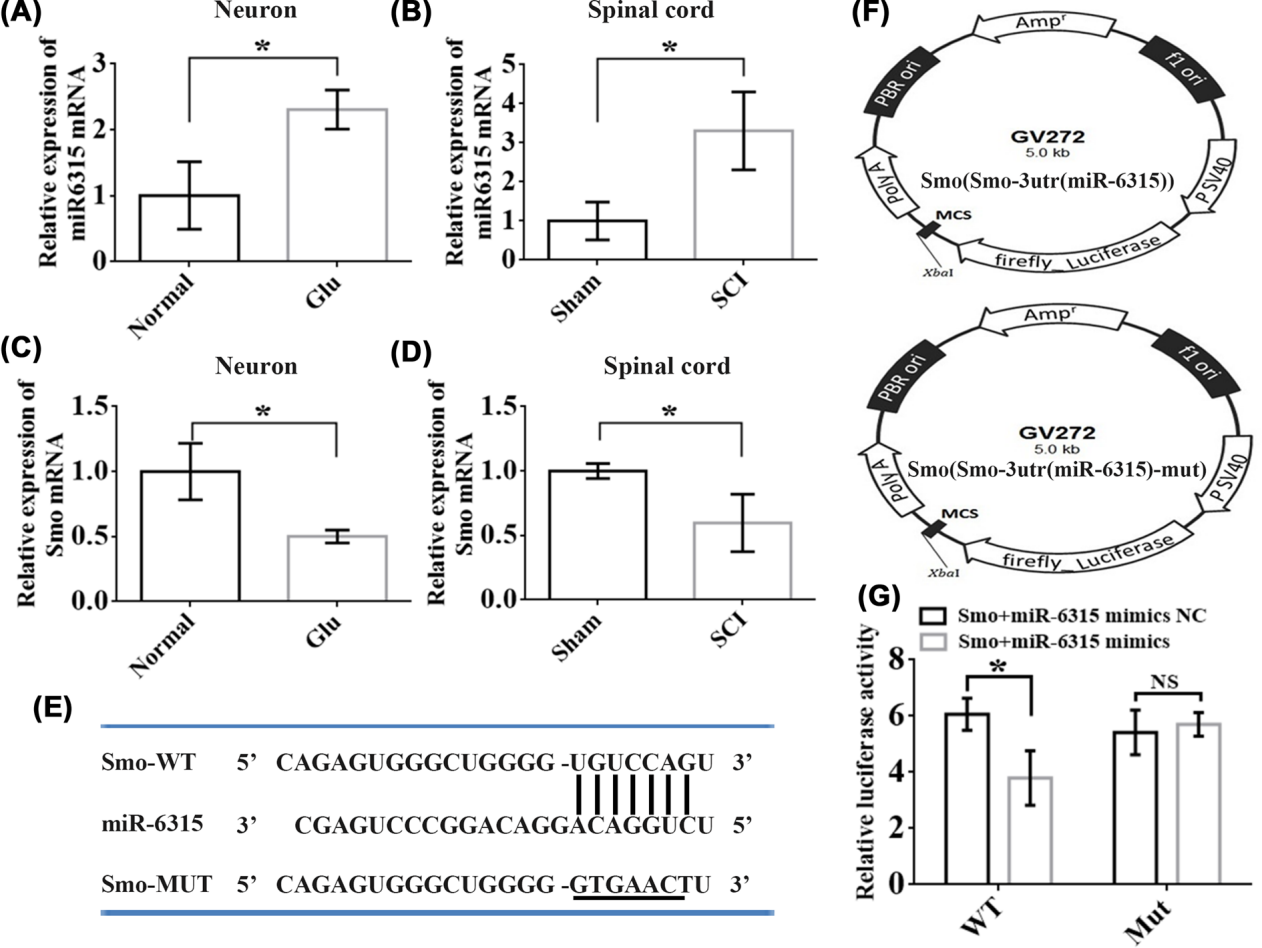
Verification of DEmiRNAs and their targeting relation (**A**) miR-6315 expression in Glu and healthy neurons. (**B**) miR-6315 expression in the spinal cord of the SCI and Sham groups. (**C**) Smo expression in Glu and normal neurons. (**D**) Smo expression in the spinal cord of the SCI and Sham groups; *n* = 3 each. (**E**) The MUT sequences of Smo 3′-UTR were prepared for luciferase reporter assay. (**F**) The constructed MUT and WT Smo luciferase plasmids. (**G**) MUT and WT Smo vector luciferase activities after cotransfection with miR-6315 vectors (*n* = 3/group). Values presented as mean ± S.D. Significance was determined using Student’s *t-*test. *P<0.05. MUT, mutant; WT, wild-type.

To verify the accuracy of whole-transcriptome sequencing, we performed qRT-PCR of significant DEmiR-6315 and Smo in spinal cord tissues and spinal cord neurons. The results of qRT-PCR revealed up-regulated miR-6315 expression (Figure 4A,B) and a significantly down-regulated Smo expression (Figure 4C,D) in spinal cord tissues and neurons after SCI and Glu treatment. These results conformed with those of the sequencing analysis, thereby validating the sequencing results. Bioinformatics analysis was performed to predict the binding site of miR-6315 in Smo (Figure 4E). Wild-type (WT) and mutant (MUT) Smo luciferase plasmids (Figure 4F) were established to verify their targeting characteristics. The binding of WT Smo and miR-6315 resulted in a significantly different signal; however, the Smo mutation abolished the signal difference, confirming that Smo has a binding site for miR-6315 (Figure 4G).

### Downregulation of miR-6315 promoted neuronal growth

**Figure 5:**
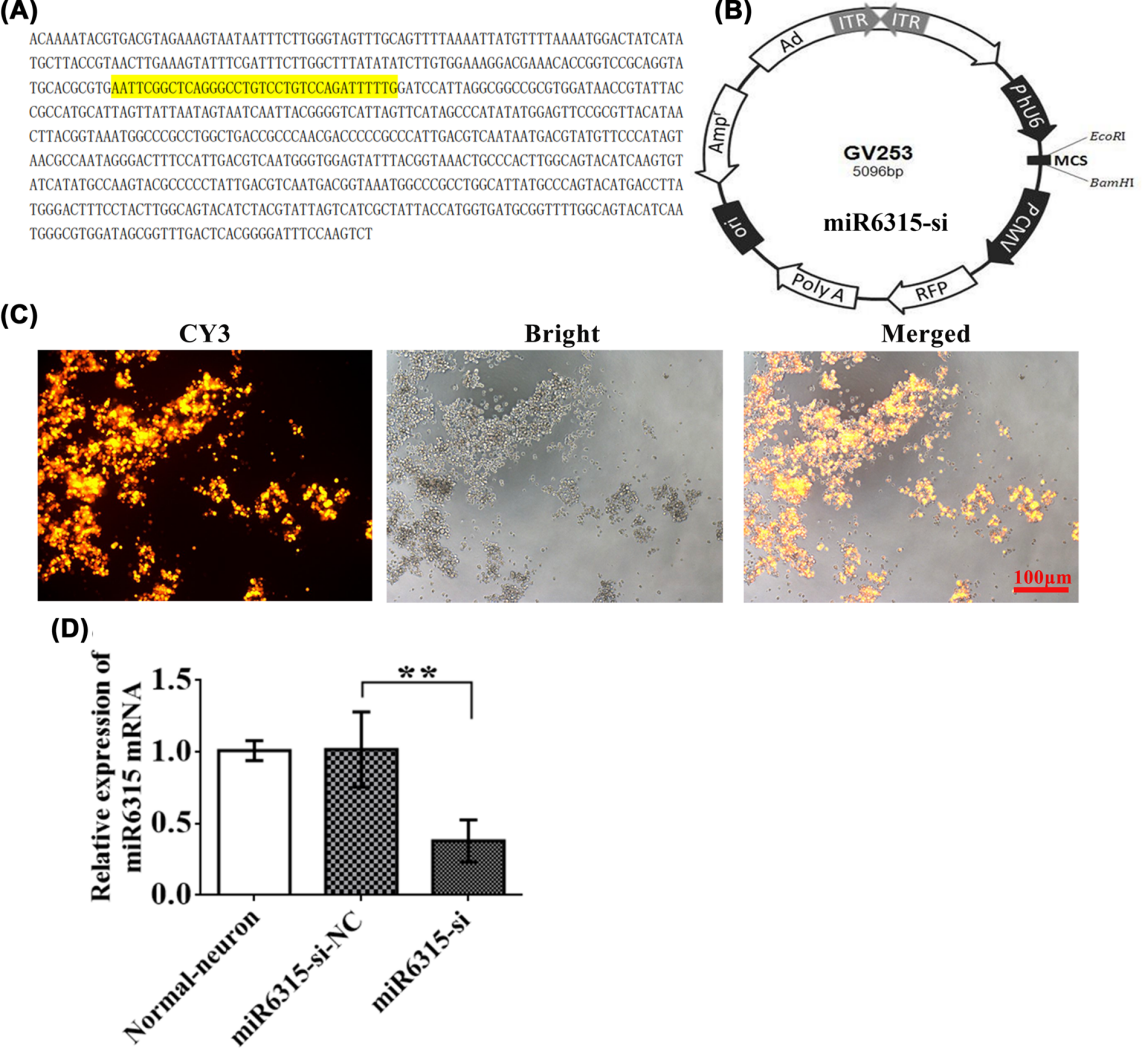
Construction and identification of the miR-6315 low-expressing adenovirus (**A**) Positive clonal sequencing results. (**B**) The vector was named GV253, which was digested with Age I/EcoR I enzymes, and molecular weights of digested products are shown. (**C**) miR-6315-si transfection into the HEK-293T cells; CY3: red color with CY3 indicates a successful transfection. Bright: a bright-field picture of HEK-293T cells. (**D**) RT-PCR determination of miR-6315. *Y* axis represents the average relative expression of miR-6315. Results are presented as mean ±SD. Ordinary one-way ANOVA was performed in combination with the LSD test. **P<0.01 vs. miR-6315-si NC group (*n* = 3/group).

To inhibit miR-6315 expression, we constructed miRNA-inhibition adenovirus. The adenoviral vector, GV253, was digested with Age I/EcoRI enzymes, and Figure 5B provides the molecular weight of plasmids in the prepared product. Positive sequencing results were obtained for the clones, which indicated the normal plasmid clones (Figure 5A). The adenovirus carrying the miR-6315 interfering (miR-6315-si) vector was transfected into the HEK-293T cell line, and the red fluorescence of CY3 indicated a successful transfection (Figure 5C). In addition, according to the qRT-PCR assay, the miR-6315 level of the miR-6315-si group markedly declined compared with the NC group (Figure 5D). The production of miR-6315-si virus efficiently transfected the spinal cord neurons and expressed the marker RNA stably *in vitro*.

**Figure 6:**
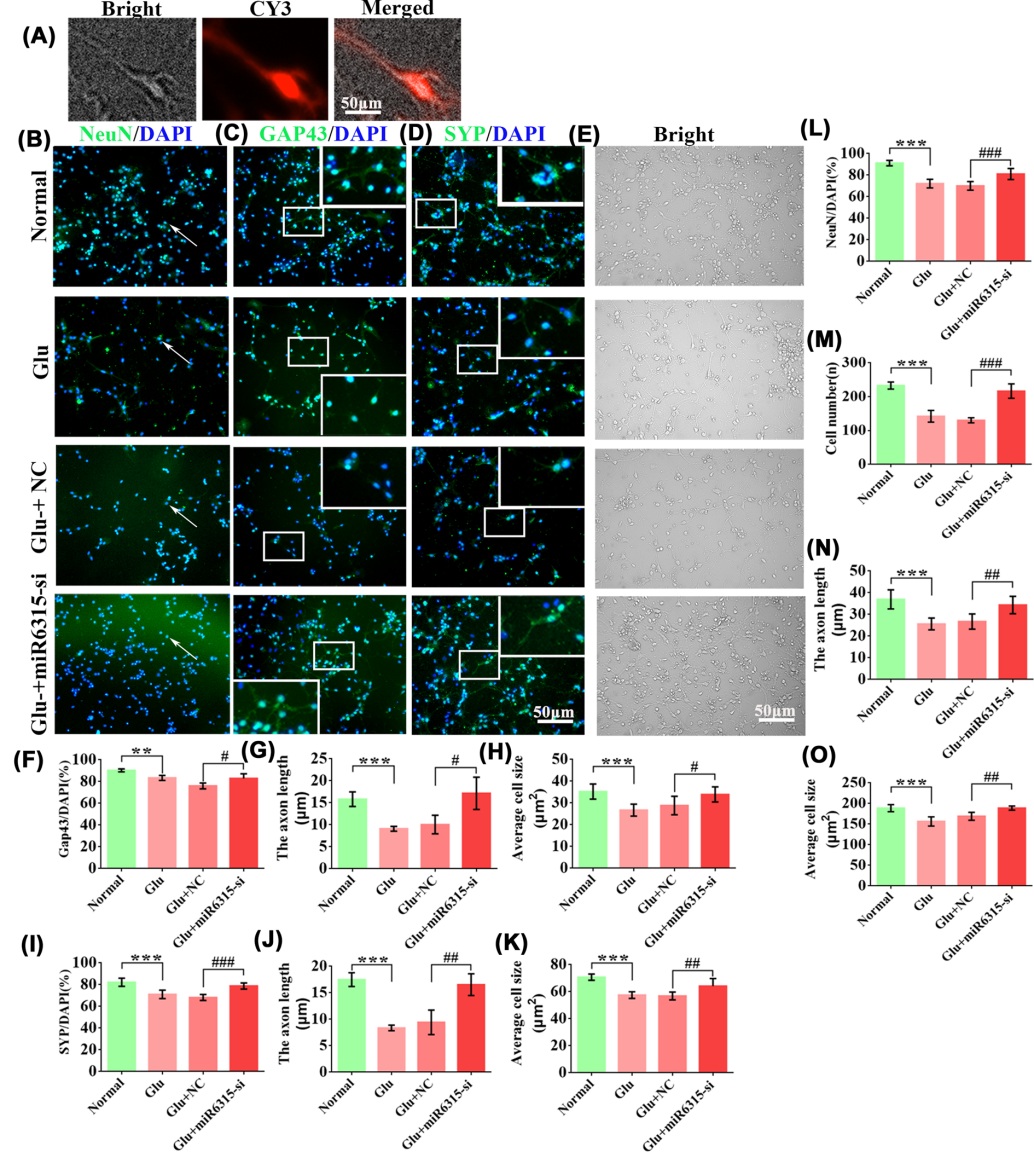
Silencing of miR-6315 improved the primary spinal cord neuron morphology and neuronal axon development (**A**) The successful transfection of miR-6315-si vectors in spinal cord neurons, represented by red fluorescence in cells; scale bar: 50 µm. (**B**) Immunofluorescence image (20X) showing neuronal cell bodies. DAPI: nuclear marker (blue); NeuN: neuronal cell bodies marker (green); bar: 50 µm. (**C**) Immunofluorescence image (20×) showing axons and neuronal cell bodies. DAPI: nuclear marker (blue); GAP43: the nerve-specific protein related to synaptic development and neuronal regeneration (green); bar: 50 µm. (**D**) Immunofluorescence image (20×) showing axons and neuronal cell bodies. DAPI: nuclear marker (blue); SYP: synaptophysin (green); bar: 50 µm. (**E**) Bright-field image (20×) revealing changes in neuronal morphology in the normal, Glu, Glu + NC vs. miR-6315-si groups; bar: 50 µm. (**F–H**) The GAP43 positivity rate, the axon length of neurons, and average cell size after Glu treatment in the normal, Glu, Glu + NC, and miR-6315-si groups were statistically analyzed; *n* = 6 each. (**I–K**) SYP positivity rate, the axon length of neurons, and average cell size after Glu treatment in the normal, Glu, Glu + NC, and miR-6315-si groups were statistically analyzed; *n* = 6 each. (**L**) NeuN positive cells after Glu treatment in the normal, Glu, Glu + NC, and miR-6315-si groups were statistically analyzed; *n* = 6 each. (**M–O**) Cell numbers, neuron axonal length, and average cell size after Glu treatment in the normal, Glu, Glu + NC, and miR-6315-si groups were statistically analyzed; *n* = 6 each. Results are presented as mean ± SD. Ordinary one-way ANOVA and LSD test were performed. **P<0.01, ***P<0.001 compared with the normal group. ^#^*P*<0.05, ^##^*P*<0.01, ^###^*P*<0.001 compared with the Glu + NC group.

To further explore miR-6315’s role in spinal cord neuron development, we transfected miR-6315-si and miR-6315-si-negative control (NC) adenoviruses in primary spinal cord neurons (Figure 6A). Simultaneously, immunofluorescence staining of NeuN, GAP-43, and SYP in spinal cord neurons under Glu treatment was performed (Figure 6B–D). Neurons expressing red fluorescence were transfected with adenovirus (Figure 6A). The NeuN staining results indicated that the miR-6315-si group increased the positive rate of NeuN after Glu treatment in primary neurons compared with the NC group (Figure 6L). The results of GAP-43 and SYP staining revealed that the 6315-si group elevated GAP43 and SYP positivity rates and reserved neuronal axon length and cell body size after Glu treatment in spinal cord neurons compared with the NC group (Figure 6F–K). In addition, Glu treatment resulted in fewer NenN/gap43/syp positive cells, shorter axons, and smaller cell body areas compared with the normal group (Figure 6F–L). We analyzed alterations in primary spinal cord neuron morphology after miR-6315-si treatment in normal and Glu conditions (Figure 6E). Glu exposure induced sparser neuronal growth, decreased axons, and smaller cell body area compared with the normal control (Figure 6M–O). The miR-6315-si group resulted in more cell numbers, longer axonal length, and larger cell body area compared with the NC group in neurons treated with Glu (Figure 6M–O). Therefore, miR-6315 silencing ameliorated the reduced cell number as well as the changes in pathological morphology after the Glu treatment; these changes manifest as cell body enlargement and longer axonal lengths in primary cortical neuronal cells.

### miR-6315 down-regulation increased spinal cord neuron migration and inhibited cell apoptosis

**Figure 7:**
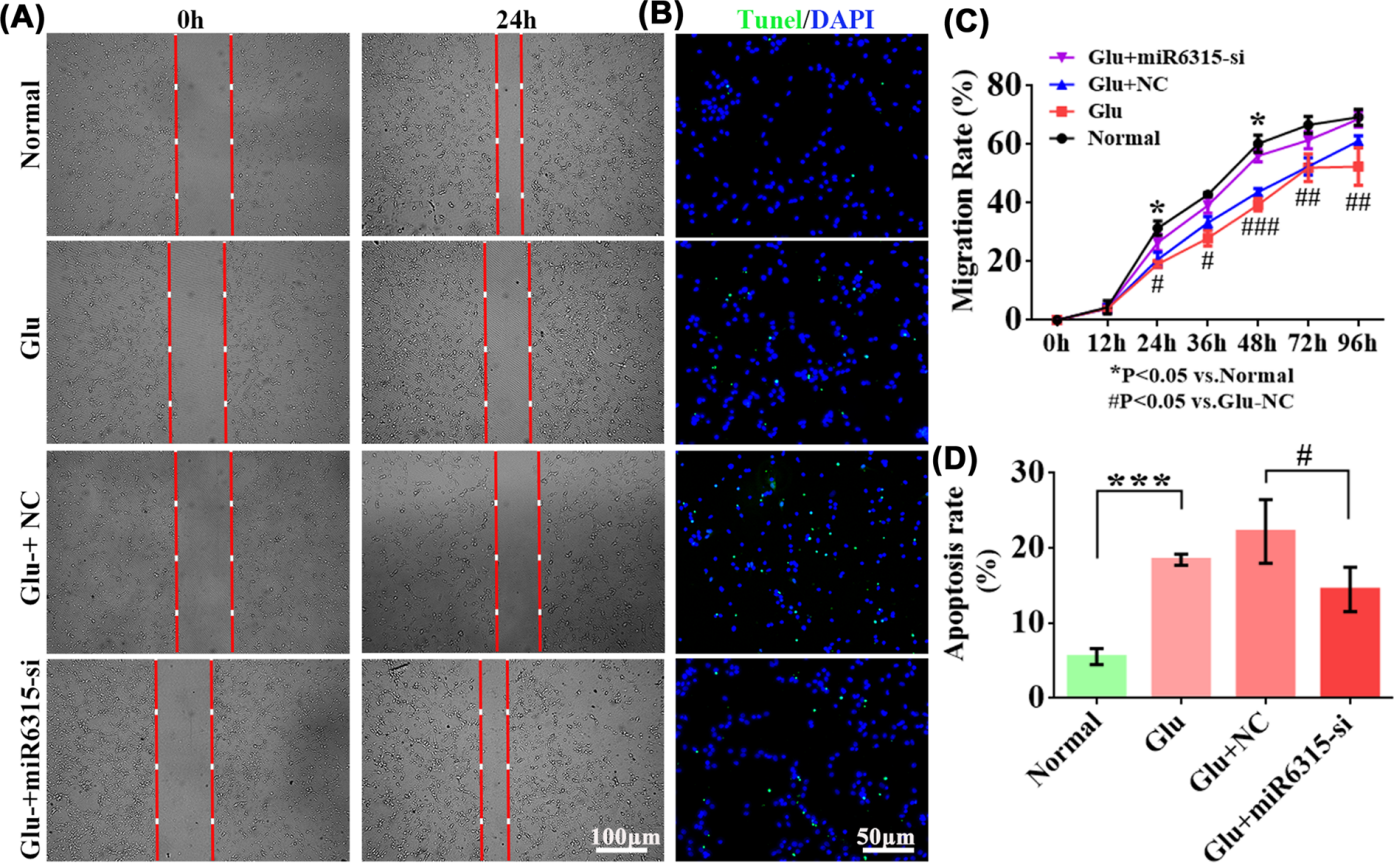
The functional assay in spinal cord neurons following miR-6315-si transfection (**A**) *In vitro* cell scratch assay in spinal cord neurons at 0 and 24 h; scale bar: 100 µm. (**B**) TUNEL staining of the spinal cord neurons following Glu treatment to detect cell apoptosis; scale bar: 50 µm. Green and blue fluorescence represent TUNEL-positive cells and nuclear staining, respectively. (**C**) Comparison of cell migration rate in each group. (**D**) TUNEL/DAPI (%) was determined to analyze cell apoptosis of Glu-treated and normal cells after adenovirus transfection 3 d following Glu treatment; *n* = 6 each. Results are presented as mean ±SD. Ordinary one-way ANOVA and LSD test were performed. *P<0.05, ***P<0.001 compared with the normal group. ^#^*P*<0.05, ^##^*P*<0.01, ^###^*P*<0.001 compared with the Glu + NC group.

The migration of spinal cord neurons is depicted in Figure 7A. After 24 h of scratching, the cell migration rate in the Glu group remarkably reduced compared with the normal group. The cell migration rate of the miR-6315-si group remarkably increased compared with the Glu + NC group (Figure 7C). The results of TUNEL staining revealed that the knockdown of miR-6315 reduced apoptotic cells after Glu treatment in spinal cord neurons (Figure 7B,D).

### miR-6315 low expression up-regulated the levels of Smo and anti-ferroptosis pathway factors in spinal cord neurons after Glu treatment

**Figure 8:**
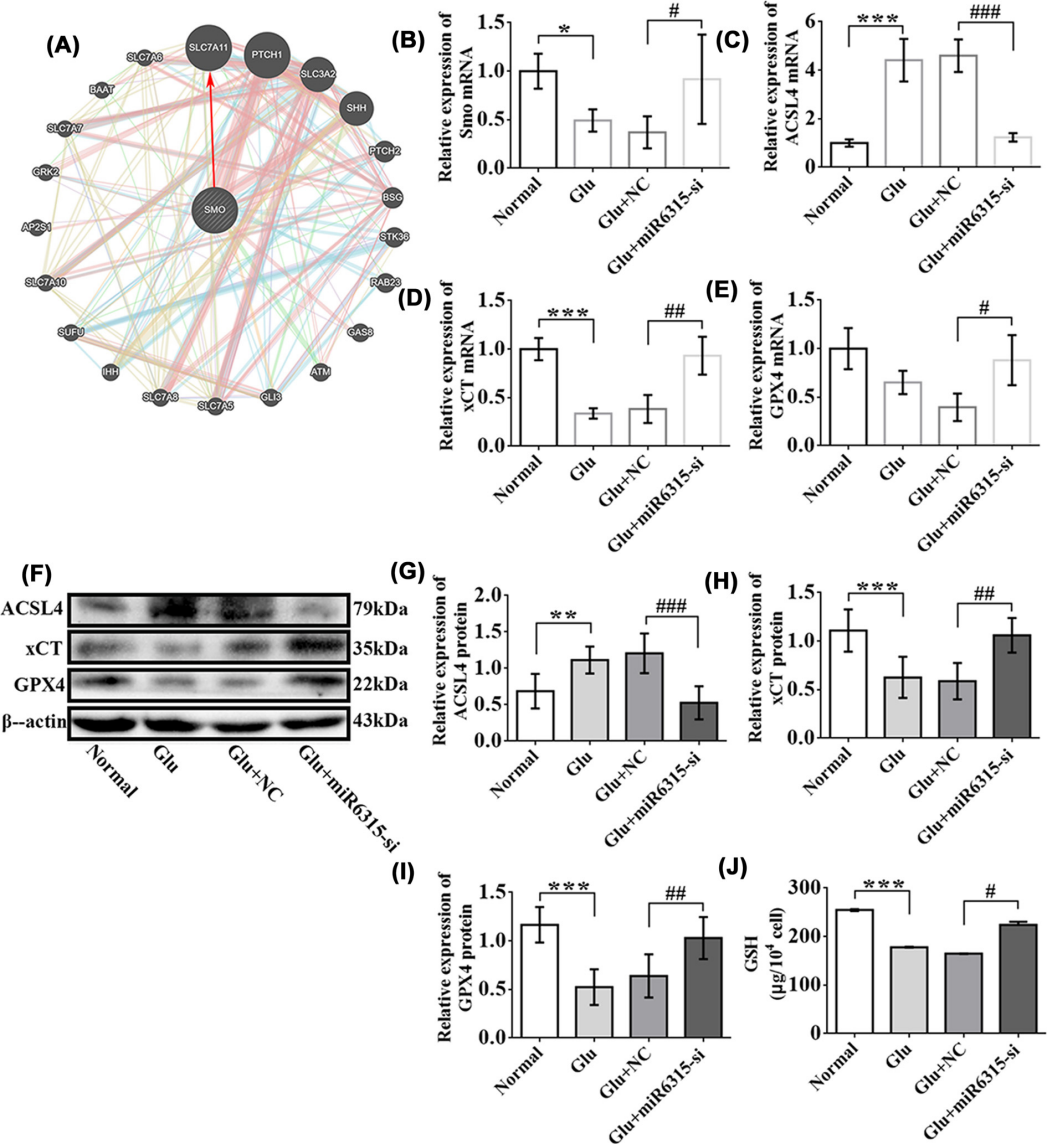
Smo and anti-ferroptosis pathway factor expression dynamics in miR-6315 silencing neurons (**A**) GeneMANIA website was used to predict Smo-associated gene network. (**B**) Smo mRNA levels of the normal, Glu, Glu + NC, and Glu + miR-6315 groups were determined. (**C**) ACSL4 mRNA levels of the normal, Glu, Glu + NC, and Glu + miR-6315 groups were determined. (**D**) xCT mRNA levels of the normal, Glu, Glu + NC, and Glu + miR-6315 were determined. (**E**) GPX4 mRNA levels of the normal, Glu, Glu + NC, and Glu + miR-6315 were determined; *n* = 3 each. (**F**) Typical immunoblots for ACSL4, xCT, GPX4, and β-actin proteins in primary spinal cord neuronal cells of the normal, Glu, Glu + NC, and Glu + miR-6315 groups after 24-h Glu treatment. (**G**) Quantitative analyses of ACSL4 protein. (**H**) Quantitative analyses of xCT protein. (**I**) Quantitative analyses of GPX4 protein; *n* = 6 each. (**J**) Quantitative analyses of GSH (*n* = 3/group). Results are presented as mean ±SD. Ordinary one-way ANOVA and LSD test were performed. *P<0.05, **P<0.01, ***P<0.001 compared with the normal group. ^#^*P*<0.05, ^##^*P*<0.01, ^###^*P*<0.001 compared with the Glu + NC group.

The results of qRT-PCR revealed that Smo levels in the miR-6315-si group increased compared with the NC group after Glu treatment in primary neurons (Figure 8B). Therefore, the role of miR-6315 in the protection of neuronal axon regeneration after knockdown may be related to Smo. The downstream signaling molecules upon which Smo exerts axon regeneration remain unknown. We used the GeneMANIA network station (http://genemania.org/) for identifying and predicting Smo downstream targets (Figure 8A). The most significantly coexpressed candidate was anti-ferroptosis pathway factor SLC7A11 (xCT). The mRNA expression of lipid peroxidation-related gene/protein (ACSL4) was down-regulated in miR-6315 knockdown and up-regulated in Glu (Figure 8C). However, the mRNA expression of anti-ferroptosis pathway factor (xCT/GPX4) was up-regulated in miR-6315 knockdown and down-regulated in Glu (Figure 8D,E). These results were validated using the WB assay (Figure 8F–I). The anti-ferroptosis pathway factor GSH test revealed that GSH expression of the miR-6315-si group obviously increased compared with the NC group after Glu treatment in primary neurons (Figure 8J). In summary, Smo expression increased during miR-6315 silencing, which may play a role in promoting axon regeneration by inhibiting ferroptosis.

### Ethological and histological analysis of miR-6315 knockdown rats

**Figure 9:**
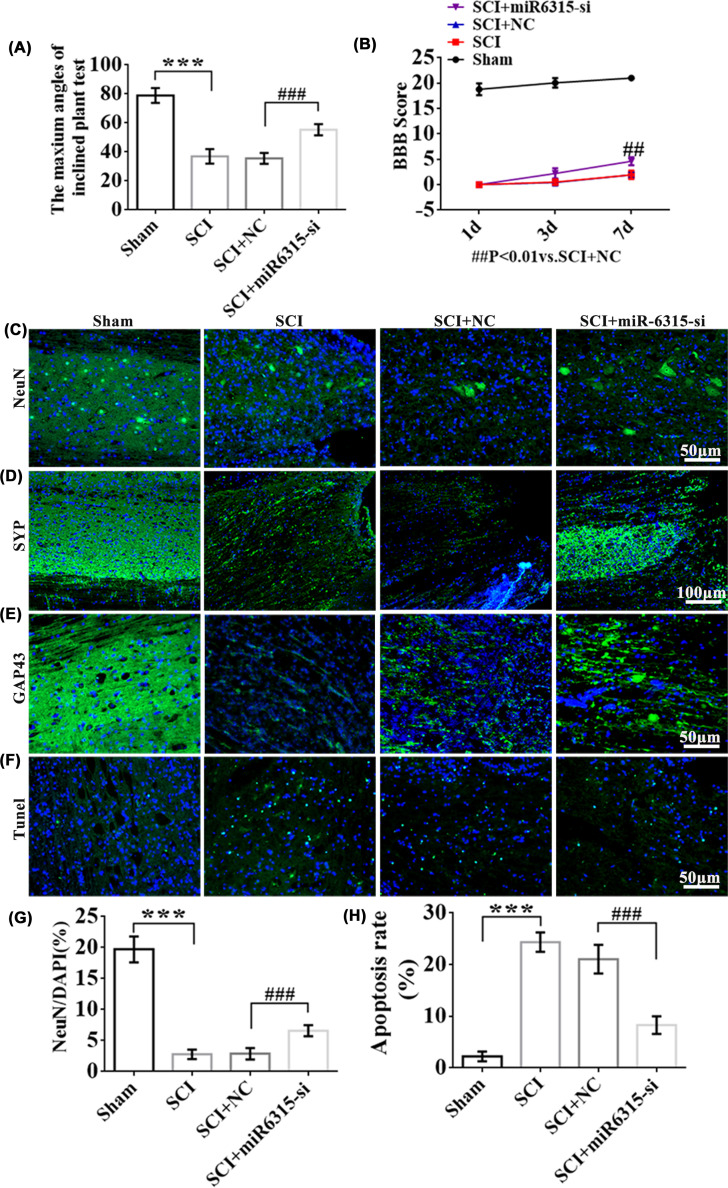
Behavioral and histological analysis of miR-6315 knockdown rats (**A**) The quantitative analysis of the maximum angle of the inclined plate in the Sham, SCI, SCI + NC, and SCI + miR-6315-si groups. (**B**) The motor functional index on day 7 post-SCI. (**C**) IHC analysis for determining NeuN (20X) expression. DAPI: nucleus marker (blue); NeuN: neuronal cell bodies marker (green); bar: 50 µm. White arrows represent NeuN^+^ cells. (**D**) IHC for determining SYP (10×) expression. DAPI: nucleus marker (blue); SYP: synaptophysin (green); bar: 100 µm. White arrows represent SYP^+^ cells. (**E**) Immunohistochemistry to determine the expression of GAP43 (20×). DAPI: nucleus marker (blue); GAP43: the nerve-specific protein related to synaptic development and neuronal regeneration (green); bar: 50 µm. White arrows represent GAP43^+^ cells. (**F**) Immunohistochemistry to determine the expression of Tunel (20×); scale bar: 50 µm. Green and blue fluorescence represent TUNEL-positive cells and nuclear staining, respectively. White arrows represent Tunel^+^ cells. (**G**) The NeuN positive cells of the Sham, SCI, SCI + NC, and SCI + miR-6315-si groups were statistically analyzed. (**H**) TUNEL/DAPI (%) values of the Sham, SCI, SCI + NC, and SCI + miR-6315-si groups were determined to analyze cell apoptosis; *n* = 6 each. Results are presented as mean ±SD. Ordinary one-way ANOVA and LSD test were performed. ***P<0.001 compared with the Sham group. ^##^*P*<0.01, ^###^*P*<0.001 compared with the SCI + NC group.

The hindlimb weight-bearing capacity of the rats was assessed using the inclined plate grasp test on day 7 post-surgery, and the maximum angle of the SCI + miR-6315-si group was remarkably elevated compared with the SCI + NC group (Figure 9A). We used BBB to assess motor function recovery in rats on days 1, 3, and 7 post-SCI. The Sham group gradually achieved a normal BBB score on day 3 post-SCI, with a score of 21. On day 7 post-SCI, the rats from the SCI + miR-6315-si group had markedly improved functions compared with the SCI + NC group (Figure 9B). The immunohistochemical (IHC) staining on day 7 post-SCI revealed that NeuN-positive cells were upregulated after miR-6315 suppression (Figure 9C,G), but the apoptosis rate was simultaneously down-regulated (Figure 9F,H). The results of GAP43 and SYP fluorescence staining revealed that GAP43 and SYP-positive cells were also up-regulated by miR-21a-5p inhibition (Figure 9D,E).

### miR-6315 low expression up-regulated the levels of Smo and anti-ferroptosis pathway factors in spinal cord tissues post-SCI

**Figure 10:**
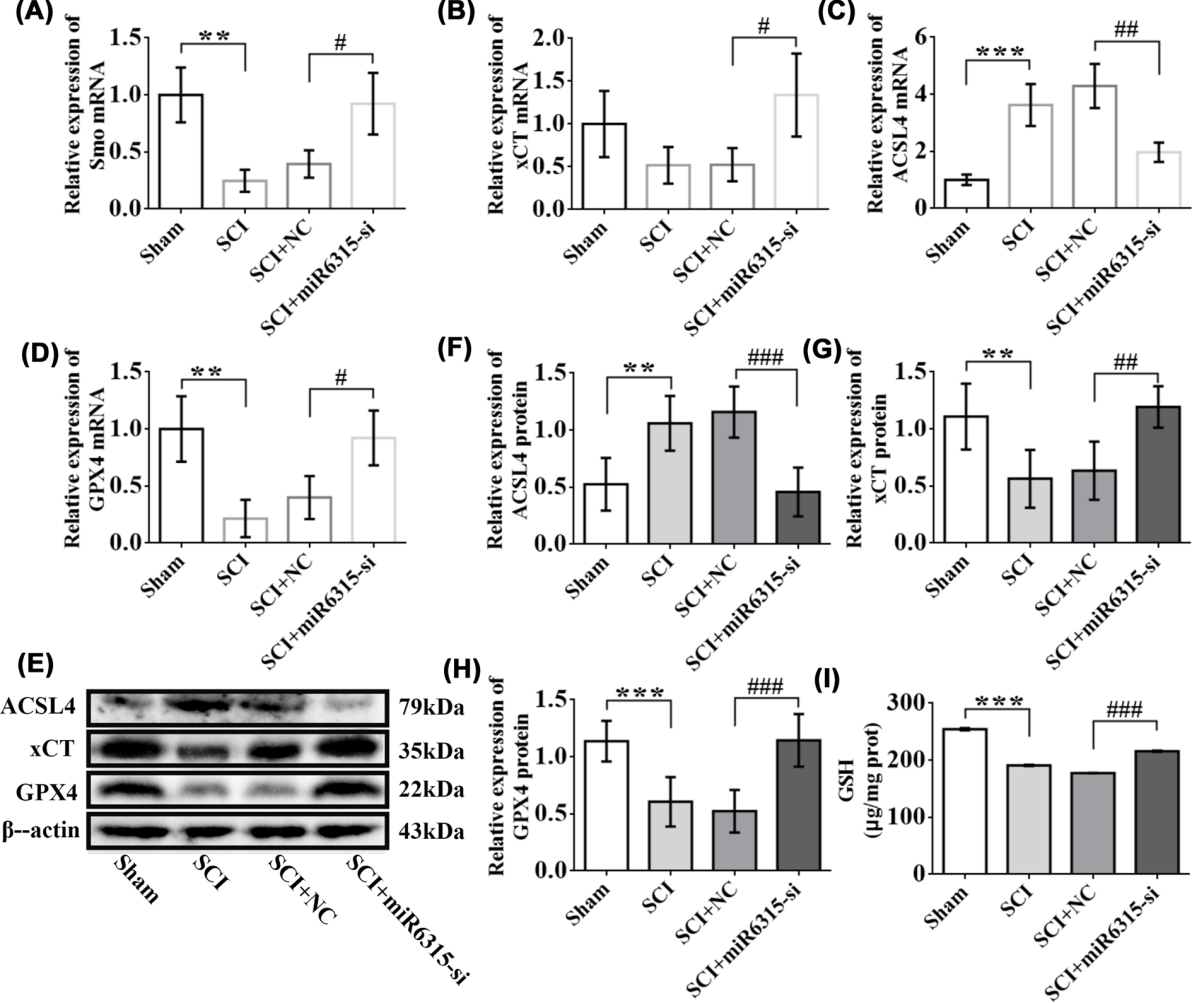
Smo and anti-ferroptosis pathway factor expression dynamics in miR-6315 knockdown rats from day 7 post-SCI (**A**) Smo mRNA, (**B**) xCT mRNA, (**C**) ACSL4 mRNA, and (**D**) GPX4 mRNA expression were evaluated in the Sham, SCI, SCI + NC, and SCI + miR-6315-si groups; *n* = 3 each. (**E**) Representative immunoblots of spinal cord tissue ACSL4, xCT, GPX4, and β-actin protein levels in the Sham, SCI, SCI + NC, and SCI + miR-6315-si groups from day 7 post-SCI. (**F**) Quantitative analyses of ACSL4 protein. (**G**) Quantitative analyses of xCT protein. (**H**) Quantitative analyses of GPX4 protein; *n* = 6 each. (**I**) Quantitative analyses of GSH (*n* = 3/group). Results are presented as mean ± SD. Ordinary one-way ANOVA and LSD test were performed. **P<0.01, ***P<0.001 compared with the Sham group. ^#^*P*<0.05, ^##^*P*<0.01, ^###^*P*<0.001 compared with the SCI + NC group.

According to the qRT-PCR assay, the Smo level of the SCI + miR-6315-si group obviously increased compared with the SCI + NC group from day 7 post-SCI (Figure 10A). The mRNA expression of lipid peroxidation-related gene/protein (ACSL4) was down-regulated in miR-6315 knockdown and up-regulated in SCI (Figure 10C). However, the mRNA expression of anti-ferroptosis pathway factor (xCT/GPX4) was up-regulated in miR-6315 knockdown and down-regulated in SCI (Figure 10B,D). WB assay was performed for further validation of these results (Figure 10E–H). The anti-ferroptosis pathway factor GSH test results indicated that GSH expression of the miR-6315-si group obviously increased compared with the NC group from day 7 post-SCI (Figure 10I). Moreover, Smo expression increased during miR-6315 silencing in spinal cord tissues 7 d post-SCI, which may promote axonal regeneration by inhibiting ferroptosis.

## Discussion

In the present study, candidate miRNAs with the most significant upregulation 24 h post-SCI were screened using sequencing techniques and bioinformatics analysis. The expression gradually decreased after 3 and 7 days post-SCI. We employed qRT-PCR and WB to examine spinal cord tissues and neurons together with behavioral experiments to examine behavior, cell growth, cell migration, and cell apoptosis. The results revealed that miRNA-6315 was up-regulated and Smo was down-regulated in spinal cord tissues with SCI and Glu-treated neurons. Low expression of miRNA-6315 could promote neuronal axonal growth, promote neuronal migration, reduce cell apoptosis, and attenuate SCI-induced motor dysfunction. This effect was associated with the up-regulation of Smo, xCT, GPX4, and GSH and the down-regulation of ACSL4. The changes in miRNAs at the SCI site were largely driven by altered changes in injured spinal cord cells because of specific neuronal cell death and migration following injury. Therefore, studying the mechanism of miRNAs in SCI would assist in the treatment of SCI through miRNA regulation.

### SCT-induced complete paralysis of lower limbs in rats

We chose female rats for the SCI study because of their shorter urethra for easy bladder emptying, relatively few complications for easy care, and no significant effect of gender factors on pathological changes and functional recovery after spinal cord injury in rats [27–30]. We constructed an SCI model, and the SCI group had a BBB score of 0 at 24 h post-SCI. The hindlimb weight-bearing capacity of the rats was assessed using the inclined plate test, and the maximum angle of the SCI group remarkably decreased compared with the Sham group; the abdomen and tail of the rats were dragged during locomotion. Nissl staining revealed that the SCI group had significantly reduced spinal cord neurons at the trauma site. SCI is a debilitating trauma causing irreversible motor and sensory impairments [31]. Injuries may be caused by vascular lesions, tumors, or viruses, apart from falls, sports injuries, and motor vehicle accidents [32].

The most common form of SCI is tissue destruction or tearing caused by external forces, resulting in altered cellular functions, such as spinal cord edema, hemorrhage, glial scarring, aggravated axonal rupture, and neuronal death [15,33]. SCI is uncurable, and specific therapeutic genes are required for correcting the damaged neural connections and creating new ones [34]. Therefore, axonal regeneration has a critical effect on SCI repair. Studies have demonstrated that the axons of most severed spinal neurons have limited regenerative capacity during early post-SCI; however, the mechanism leading to the termination of initial regenerative activities of axons remains unclear. Some scholars believe that the termination of regenerative activities may be due to the existence of a microenvironment that inhibits growth cones [9,14]. Therefore, SCI should be treated by improving the injured spinal microenvironment and minimizing secondary injury, thereby inhibiting further neuronal loss and stimulating axonal regeneration.

### Knockdown of miR-6315 inhibited apoptosis, promoted neuronal survival and migration, and improved motor function post-SCI

Several studies have indicated that the miRNA/mRNA axis has a critical effect on the pathophysiology of the nervous system [20,21,35]. We identified DEmiRNAs and DEmRNAs post-SCI by using whole-transcriptome sequencing. We performed bioinformatics analysis and screened out a miRNA, miRNA-6315, that was significantly up-regulated at 24 h of post-SCI and whose level gradually decreased at 3 and 7 days after SCI. Therefore, miRNA-6315 may affect the repair process of SCI.

miRNAs modulate the expression of several genes after SCI at the post-transcriptional level, thereby regulating the cell state and function. Therefore, the in-depth study of miRNAs can facilitate the treatment and prognosis prediction of SCI [36,37]. A study analyzed miRNAs for elucidating changes in mTOR pathway activation following T10 SCT [38]. Another study indicated that miR-6315 may participate in osteogenesis/adipogenesis by regulating the TGF-β/Smad2 signaling pathway [39]; however, no study has focused on miR-6315 in SCI. The *in vivo* and *in vitro* experiments revealed that the expression of miR-6315 was significantly up-regulated after SCI or Glu treatment, and the results were consistent with sequencing results. In addition, the construction of miR-6315 low expression adenovirus vector, NeuN, GAP43, SYP, TUNEL staining, and the scratch test revealed that miR-6315 knockdown facilitates neurite outgrowth and cell migration while attenuating cellular apoptosis. Therefore, cell recovery may be affected by the down-regulation of miR-6315 expression. Furthermore, miR-6315 was knocked down in SCI rats to examine the recovery of motor function. Because secondary injury predominates following the acute phase (3 days after SCI), we selected 7 days after SCI as the final observation period to better evaluate the effect of miR-6315 knockdown treatment. According to the results of the BBB score and the slant plate test, the recovery of motor function in the miR-6315 knockdown group increased relative to the NC group post-SCI. Immunohistochemical analysis of the miR-6315 knockdown group exhibited increased neurite regeneration and decreased apoptosis after SCI. in summary, miR-6315 has a major effect on the post-SCI repair.

### miR-6315 knockdown up-regulated the levels of Smo and anti-ferroptosis pathway factors to improve motor function and promote neuronal repair

Mature miRNAs can combine with 3′-UTRs in target mRNAs, resulting in repression or degradation of mRNA translational, causing changes in cell function and protein structure [40]. We used GO and KEGG enrichment analysis to discover the following three mRNA candidates enriched in the axonal growth cone pathway: RTN4R, GPM6A, Smo. We predicted that Smo has a binding site for miR-6315 by using the Ensembl database and Miranda software. The miR-6315/Smo axis has a critical effect on the regulation of pathological changes post-SCI. Subsequently, the interaction site between miR-6315 and Smo was preliminarily verified in PC12 cells by using the dual-luciferase reporter assay. Smo expression reduces post-SCI in the spinal cord neuronal cells and tissues. However, the expression of Smo was up-regulated after 6315 knockdown post-SCI and after Glu treatment.

Smo belongs to the Frizzled family of seven transmembrane domain proteins [41]. Studies have demonstrated that the spinal cord exhibits abnormal growth in the presence of hyperactive Smo [42]. However, the indirect role of Smo-dependent spinal cord regeneration in neural stem cells through spinal cord-related pathways remains unclear [43]. A study demonstrated that the inhibition of Smo downstream factor PKA had a smaller effect on the regeneration of spinal cords than Smo suppression, and spinal cord regeneration was almost completely blocked when both Smo and PKA were inhibited [44]. Therefore, PKA may not be a linear downstream factor of the Smo-mediated pathway [42]. The downstream signaling molecules on which Smo exerts axon regeneration remain unclear.

We predicted the Smo-associated genes by using GeneMANIA and the anti-ferroptosis pathway factor SLC7A11 (xCT). xCT, the light chain subunit of System xc-, is an antiporter that imports cystine while releasing glutamate [45]. Stimulating anti-ferroptosis pathways, namely SLC7A11/glutathione/glutathione peroxidase 4 (xCT/GSH/GPX4), could improve the survival of damaged spinal cord neurons [46] and enhance their functional recovery post-SCI [47]. However, it remains unclear whether Smo has a crucial effect on axonal regeneration by promoting anti-ferroptosis pathways (xCT/GSH/GPX4) and inhibiting ferroptosis. WB and qRT-PCR assays were conducted to detect differential levels of xCT, GSH, GPX4, and ACSL4 (lipid peroxidation-related gene) post-SCI. The results revealed that xCT/GSH/GPX4 expression was relatively low, and ACSL4 was up-regulated post-SCI and after Glu treatment in spinal cord tissues and neurons. However, after SCI and Glu treatment, the expression levels of xCT/GSH/GPX4 increased, and ACSL4 decreased after the 6315 knockdown. Generally, miR-6315 is related to motor function improvement and neuronal repair after SCI through the up-regulation of Smo expression and anti-ferroptosis pathway factors (xCT/GSH/GPX4).

## Conclusion

In conclusion, miR-6315 may have a critical effect on SCI repair. The neuroprotective effect of miR-6315 silencing was associated with the regulation of Smo and anti-ferroptosis pathway factors xCT, GSH, and GPX4. Therefore, miR-6315 can be used as a target to evaluate the regulation of axonal regeneration and ferroptosis and enhance SCI repair.

## Institutional Review Board Statement

All of the assays were conducted following Guide for the Care and Use of Laboratory Animals from US National Institutes of Health. The animal study protocols were reviewed and gained approval from Animal Care and Welfare Committee of Kunming Medical University (approval No.: kmmu 20211409).

## Data Availability

The miRNA sequencing data have been deposited in the SRA database via NCBI with the BioProject ID PRJNA916863. The datasets used and/or analyzed in this study will be made available on request.

## Abbreviations

BBB: Basso–Beattie–Bresnahan
GSH: glutathione
HTS: high-throughput sequencing
IF: immunofluorescence
PBS: phosphate- buffered saline
PFA: paraformaldehyde
SCI: spinal cord injury
SPF: specific pathogen-free
WTS: whole transcriptome sequencing
